# Botulinum Neurotoxins in Central Nervous System: An Overview from Animal Models to Human Therapy

**DOI:** 10.3390/toxins13110751

**Published:** 2021-10-22

**Authors:** Siro Luvisetto

**Affiliations:** National Research Council of Italy-CNR, Institute of Biochemistry and Cell Biology (IBBC), Via Ercole Ramarini 32, Monterotondo Scalo, 00015 Roma, Italy; siro.luvisetto@cnr.it

**Keywords:** botulinum, peripheral nervous system, central nervous system, animal models, humans

## Abstract

Botulinum neurotoxins (BoNTs) are potent inhibitors of synaptic vesicle fusion and transmitter release. The natural target of BoNTs is the peripheral neuromuscular junction (NMJ) where, by blocking the release of acetylcholine (ACh), they functionally denervate muscles and alter muscle tone. This leads them to be an excellent drug for the therapy of muscle hyperactivity disorders, such as dystonia, spasticity, and many other movement disorders. BoNTs are also effective in inhibiting both the release of ACh at sites other than NMJ and the release of neurotransmitters other than ACh. Furthermore, much evidence shows that BoNTs can act not only on the peripheral nervous system (PNS), but also on the central nervous system (CNS). Under this view, central changes may result either from sensory input from the PNS, from retrograde transport of BoNTs, or from direct injection of BoNTs into the CNS. The aim of this review is to give an update on available data, both from animal models or human studies, which suggest or confirm central alterations induced by peripheral or central BoNTs treatment. The data will be discussed with particular attention to the possible therapeutic applications to pathological conditions and degenerative diseases of the CNS.

## 1. Introduction

Botulinum neurotoxins (BoNTs) are toxins produced by the bacteria *Clostridium botulinum* in many variants of seven well-characterized serotypes [1], named from A to G, and other serotypes, defined as H, FA, X, or Wo, whose existence as new serotypes is still debated [2,3,4]. In the following text, the different types of BoNTs will be reported with the acronym BoNT/Y, where Y stands for A to G, regardless of which toxin, from a laboratory or commercial product, was effectively used. If subtype is not specified, the acronym BoNTs will be still used in generic way.

BoNTs exert their canonical action by entering nerve endings at neuromuscular junction (NMJ) where, by cleaving soluble N-ethylmaleimide-sensitive factor-attachment protein receptors (SNARE) proteins, they prevent the vesicular release of acetylcholine (ACh) from the synaptic terminal, causing muscle relaxation and flaccid paralysis [5,6]. Each serotype has different SNARE proteins target: BoNT/A, C, and E cleave the 25 kDa synaptosomal-associated protein (SNAP25); BoNT/B, D, F, and G cleave the vesicle associated membrane protein (VAMP)/synaptobrevin; BoNT/C also cleaves the sintaxin. The muscle relaxation induced by BoNTs underlies their use as elective drugs for the therapy of many neurological diseases that depend etiologically on excessive releases of ACh [7,8]. Nowadays, a peripheral intramuscular (i.m.) injection of non-toxic quantities of BoNT/A or B, constitutes a therapeutical treatment of diseases characterized by excessive muscle contractions, with new emerging uses [9,10,11] continuously expanding the FDA approved indications [12]. Moreover, depending on the target tissue, BoNTs can block not only the cholinergic neuromuscular release but also cholinergic autonomic innervation of exocrine glands. Further, it is well known that the inhibition of synaptic release is not limited to release of ACh, but also to release of other neurotransmitters, mainly excitatory, such as glutamate, CGRP, and substance P. This makes BoNTs an excellent therapeutic treatment not only for muscle hyperactivity, but also for conditions dependent from release of excitatory neurotransmitters, such as, for example, urological disorders [13] or chronic painful conditions [14] including headache/migraine [15,16] and painful musculoskeletal [17] or neuropathic conditions [18].

**Figure 1:**
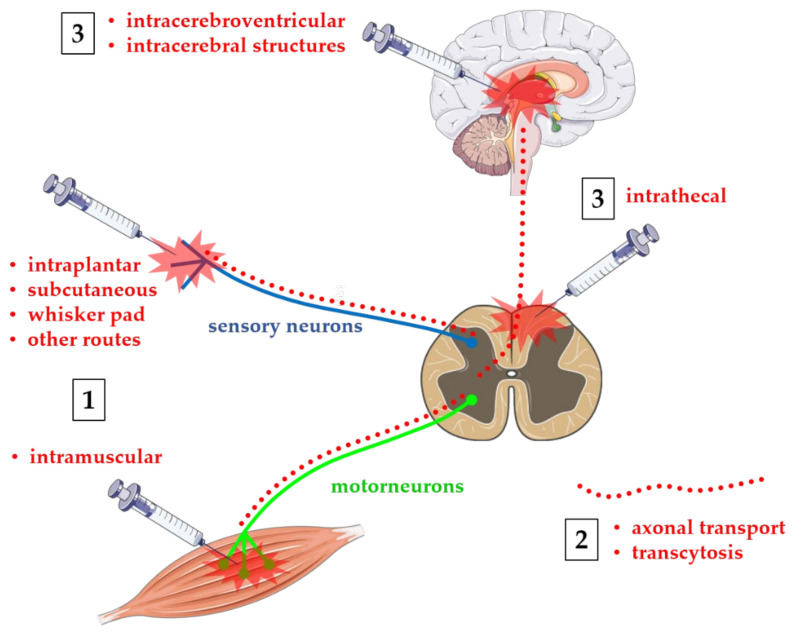
Routes of administration of BoNTs. Toxins are administered at different peripheral or central sites depending on their use in humans or animals: (**1**) peripheral BoNTs are normally injected intramuscular in humans/animals, or intraplantar, subcutaneous, on the whisker pad, or other routes in animal models; (**2**) peripheral BoNTs can be transported from the site of injection to CNS via retrograde axonal transport and transcytosis (red dotted lines) from motoneurons and/or sensory neurons toward central neurons; and (**3**) BoNTs may be administered directly to CNS via intrathecal injection at spinal cord or intracerebral level in animal models of spinal cord injuries or neurodegenerative diseases. Figure was produced by using free images taken from Servier Medical Art (http://smart.servier.com, accessed on 8 September 2021), a service to medicine provided by Les Laboratoires Servier (http://www.servier.com, accessed on 8 September 2021).

Although the clinical benefits of BoNTs injections mainly depend on their action at level of peripheral nervous system (PNS), and much basic and clinical research has been largely focused on the peripheral effects of BoNTs, many evidence from animal models and human study also confirmed BoNTs action at the level of the central nervous system (CNS). It is well established that indirect central effects can be produced by peripherally injected BoNTs through peripheral mechanisms of alteration of central sensorimotor integration [19,20]. In many cases, this may constitute a secondary beneficial effect which helps to reinforce primary peripheral effect. Alternatively, a direct central effect may be a consequence of retrograde axonal transport of BoNTs from the injection site to the central structures [21]. Obviously, it should still be noted that, as BoNTs are agents that cause botulism, the axonal transport from the PNS to the CNS can be an undesirable adverse effect, leading to possible lethal outcomes of peripheral administration. Finally, much research has focused on characterizing the effects of BoNTs directly administered to CNS, both at spinal intrathecal (i.th.) level or intracerebral in specific brain structures. As an example, much evidence has been produced, mainly in animal models for safety reasons, in favor of a possible role of BoNTs in the treatment of spinal cord injuries or cerebral neurodegenerative processes [22,23,24]. Possible routes of administration of BoNTs are depicted in Figure 1. The purpose of this review is to provide an update on past and current research on direct and indirect effects of BoNT on the CNS.

## 2. Indirect Central Effects following Peripheral Injection of BoNTs

Support for the indirect central effects of BoNTs originates from the observation that not all clinical effects of peripheral i.m. injections can be explained by the exclusive action of BoNTs on peripheral nerve terminals. Often, the therapeutic benefit exceeds the duration of neurotoxin-induced peripheral neuroparalysis. Many studies have suggested that BoNTs may indirectly influence the functional organization of the CNS through an alteration mechanism induced by altered peripheral inputs. [19]. The first evidence supporting indirect central effects of BoNTs came from experimental studies with BoNT/A in rats. When injected into the jaw muscles of rats, BoNT/A induced blockage of the γ-motoneuronal endings, reducing the spindle afferent discharge [25]. Morphological study confirmed that, when injected into skeletal muscles, BoNT/A acts at the intrafusal as well the extrafusal NMJ, causing fiber atrophy and spread of Ach staining in end-plates, resulting in denervation of both extrafusal and intrafusal fibers [26]. At the spinal level, the inhibition of motoneuronal functionality, with reflex inhibition and suppression of input from afferent fibers, results in various effects on CNS [27]. Briefly, peripheral deafferentation at the injection site produces alterations in presynaptic input from the neuromuscular connection to the γ-motoneuronal endings and intrafusal muscle fibers, modifying the excitability of spinal pathways and causing alterations of motor maps at the cortical level. The block of the afferent inflow of the spindle directed to the spinal motor neurons therefore interferes with the spinal circuits, producing possible alterations in the brain stem and cortical circuits, causing an alteration of cortical excitability and a plasticity/reorganization of various cortical areas, including thalamus and sensorimotor cortex [28]. As basal ganglia receive projection from thalamus and sensorimotor cortex, activity of basal ganglia is also altered by BoNTs-induced changes in motor afferent feedback. In another way, the presynaptic blockade of the neuromuscular connection between α-motoneuronal endings and extrafusal muscle fibers may induce plastic adaptive reorganization of the motoneuron as well [21,29].

Cortical effects following peripheral BoNTs treatment in humans were demonstrated in many functional studies using neurophysiological techniques [30]. Among others, studies of functional magnetic resonance imaging (fMRI) during motor task [31,32], performed mainly in post-stroke or dystonic patients, gave considerable help to understand which cortical areas could be affected during therapy with i.m. injection of BoNT/A. Due to the vastness of the topic and the large amount of studies in the literature, only a selection of the most significant studies will be briefly summarized in the next paragraphs (for a more detailed review, see Hok et al. [33]).

Senkarova et al. [34] performed an fMRI study to localize the changes in cerebral cortex activation in a small group of patients with post-stroke upper limb spasticity treated with BoNT/A (50 U of Botox^®^ for each muscle in four muscles of the hand). They found a relationship between dynamic changes in hand movement-related brain activation and motor improvement induced by BoNT/A treatment. In particular, a relative decrease of activation in the posterior cingulate/precuneus region after BoNT/A treatment was evident when compared with that seen in the patients prior to the treatment. In another study, Manganotti et al. [35] analyzed post-stroke patients with hemiparesis and associated upper-limb hypertonus. Before BoNT/A (total dose of 200 U of Botox^®^ in muscles of the hand), movements of paretic hand evoked a wide bilateral activation in the sensorimotor areas, in the supplementary motor area, and cerebellum. After BoNT/A, the blood oxygenation level-dependent activation decreased in ipsilateral and contralateral motor areas, in ipsilateral cerebellar regions, and in the supplementary motor area. Similar results in fMRI activity in sensory motor cortex, secondary somatosensory, and supplementary motor areas were obtained by Diserens et al. [36], who found that repetitive arm cycling training enhanced the antispastic effect of i.m. BoNT/A (25–100 of U Botox^®^ in various arm and hand muscles depending on patients) in postischemic spastic hemiparesis. Prior to BoNT/A treatment, Veverka et al. [37,38] found extensive task-related fMRI activation of bilateral frontoparietal sensorimotor cortical areas, anterior cingulate gyrus, pallidum, thalamus, and cerebellum in patients with upper limb post-stroke spasticity. Four and eleven weeks after BoNT/A treatment (50 U of Botox^®^ in various hand muscles depending on patients) fMRI activation was strongly reduced. Significant decrease of fMRI activation was located also in areas outside the classical sensorimotor system, namely, ipsilateral to lesioned lateral occipital cortex, supramarginal gyrus, and precuneus cortex. Tomasova et al. [39], in a group of patients suffering for hemiparesis and distal arm spasticity due to chronic ischemic stroke, evidenced a relief of post-stroke arm spasticity after BoNT/A injection (50 U of Botox^®^ for each muscle in various muscles). Antispastic effects of BoNTA/A correlated with changes at levels of cortical sensorimotor system and of prefrontal cortex. In another interventional study, Bergfeldt et al. [40] analyzed chronic stroke patients with right-sided hand paresis and spasticity. Peripheral effects after focal spasticity management with i.m. injection of BoNT/A (total dose of 120–390 U Botox^®^ in various group of hand muscles depending from patients) were assessed by functional tests paralleled by assessment of brain activity recorded by fMRI technique during standardized motor task focusing on the motor and pre-motor cortex. At baseline, before the therapy with BoNT/A, brain activity in the motor and pre-motor cortex, especially on the ipsilateral hemisphere, of stroke patients was 2–4.5 times higher compared with healthy subjects. After therapy with BoNT/A, there was a significant reduction in spasticity and functional improvement with, in parallel, a larger decrease in the ipsilateral and a minor decrease in the contralateral brain activity.

With fMRI during skilled hand motor task, Opavsky et al. [41] examined patients with cervical dystonia, before and four weeks after BoNT/A application to cervical neck muscles (25 U of Botox^®^). fMRI data demonstrated reduced extent of hand movement-related cortical activation in dystonic patient, together with extensive changes in contralateral secondary somatosensory cortex, and altered activation of the ipsilateral supplementary motor area and dorsal premotor cortex [41]. Activation in primary and secondary somatosensory cortex was also analyzed by Dresel et al. [42] in patients affected by idiopathic orofacial dystonia. Authors found that, although BoNT/A (185 ± 66 U of Dysport^®^ into periorbital region) did not modulate the impaired cortical activation, it reduced the activation of the thalamus and contralateral putamen during forehead stimulation. This highlights an indirect effect of BoNT/A on these sensorimotor circuits with critical functional change within the basal ganglia-thalamocortical loops. Using resting state fMRI, Delnooz et al. [43,44] evidenced altered functional brain connectivity in cervical dystonia patients. Functional MRI was repeated before and few weeks after BoNT/A injections, at doses not specified, to evidence whether connectivity abnormalities were restored. Cervical dystonia patients showed both increased and decreased connectivity in sensorimotor and in executive control network, comprising selected regions of premotor cortex, anterior cingulate cortex, parietal cortex, superior parietal lobule, middle temporal gyrus area, and in a distributed network comprising the supplementary motor area, primary sensorimotor cortex, and secondary somatosensory cortex. Brodoehl et al. [45] also reported alterations in local brain function and connectivity in cervical dystonia, with increased connectivity between the basal ganglia and the sensorimotor network, together with loss of functions in putamen, thalamus, and somatosensory cortex. They observed a partial normalization of brain activity and connectivity between basal ganglia and sensorimotor cortex after BoNT/A treatment (see [45] for details on type of toxin, doses, and injection protocols).

Another significant demonstration of indirect central effects of the peripheral toxin comes from Nevrly et al. [46]. In cervical dystonic patients, BoNT/A (25 U of Botox^®^) significantly increased the finger movement-induced fMRI activation of several brain areas, including bilateral (primary and secondary somatosensory cortex; superior and inferior parietal lobule; supplementary motor and premotor cortex; and anterior cingulate cortex), contralateral (primary motor cortex), and ipsilateral (thalamus; insula; putamen) activation. fMRI activation was also observed in the central part of cerebellum, close to the vermis. Changes in cerebellar activation after spasticity treatment with BoNT/A were also observed by Chang et al. [47] and Hok et al. [48] who provided evidence for modulation of cerebello-cortical connectivity in cervical dystonic patients treated with BoNT/A. Moreover, Li et al. [49] reported an fMRI study on patients affected by botulism after cosmetic application of BoNT/A (dose of toxin not specified). Compared with the controls, patients with botulism exhibited significantly abnormal spontaneous activity in the parahippocampal gyrus and in the cerebellum, both at anterior and posterior lobe.

Overall, the studies briefly summarized here demonstrate a correlation between the peripheral effects, i.e., muscle relaxation after BoNT/A injection, and indirect central effects. Furthermore, what emerges from these and other studies [33,50] is that the central effects induced by the peripheral injection of BoNT/A are not limited to the cortical and subcortical representations of the treated muscles, but they extend beyond the circuits that underlie the control of the affected parts of the body.

Interesting, and for certain aspects unusual, evidence of indirect effects of BoNTs on CNS has been presented by Yesudhas et al. [51]. Authors found that i.m.-injection of a mild dose of BoNT/A (1 U/Kg of Botox^®^) in adult mice improves learning and memory, tested with Morris water maze and object recognition tests, in association with increased circulating platelets and enhanced hippocampal plasticity, evidenced by enhanced density of pyramidal neurons. The mechanism responsible for these effects is not clear; however, it is worth noting that circulating platelets are a source of brain-derived neurotrophic factor, one of the major determinants and mediators of neuroplasticity, including learning and memory.

## 3. Axonal Transport after Peripheral Injection of BoNTs

Cortical reorganization following a decreased peripheral sensory input is not the only mechanism explaining centrally mediated motor recovery after peripheral injection of BoNTs. The central effects may arise from retrograde axonal transport of BoNTs in the spinal cord and their transcytosis from motor neurons to secondary spinal neurons. Historically, the first evidence of axonal transport of BoNTs came from pioneering studies on rats from Caleo and coworkers [52,53,54,55,56]. The initial observation was the finding cleaved SNAP25 (cl-SNAP25) in facial motoneuron projecting to the whisker muscle after injection of BoNT/A (0.3 μL of 3 nM solution of laboratory prepared toxin) in whisker pad [52]. In another experiment, significant levels of cl-SNAP25 were detected in the tectum after BoNT/A (0.4 μL of 1–3 nM solutions of laboratory prepared toxin) delivery into the eye [53]. Blockage of BoNT/A propagation by co-injection of colchicine ruled out a systemic spread of the toxin [53]. Evidence of transcytosis in rat visual systems has been obtained by Restani et al. [54]. The authors showed that BoNT/A (0.3 μL of 2 nM solution of laboratory prepared toxin) axonally propagates at least two synapses away from the injection site, as evidenced by the expression of cl-SNAP25 in photoreceptors and bipolar rod cells after injection of BoNT/A into the tectum. Long distance transport of BoNT/A was confirmed by detection of cl-SNAP25 in spinal cord motor neurons after injection of BoNT/A (0.5 μL of 1 nM solution of laboratory prepared toxin) into the hind leg muscles of adult rats [55]. Another evidence demonstrating trans-synaptic migration of BoNT/A (75 pg/rat; 7.5 pg/mouse) into secondary synapses, came from the observation of cl-SNAP25 within the facial motor nucleus, after toxin application into the whisker pad muscles is prevented by BoNT/A-specific antitoxin applied into the lateral ventricles or cisterna magna [56]. Furthermore, trafficking of BoNT/A and D were demonstrated not only in primary motoneurons, but also in central neurons from in vitro study using microfluidic devices [57].

It should be noted that data previously described were based on the detection of cl-SNAP25 as assay of BoNT/A trafficking. As BoNT/A is a proteolytical enzyme, and very low amounts of toxin molecules can proteolyze a large number of SNAP25, providing a dramatic amplifying effect, this was considered a reliable tool to monitor the presence of active BoNT/A in vivo. However, retrograde transport of BoNT/A was also confirmed by directly measuring the distribution of radiolabeled BoNT/A, with γ-emitting radionuclide technetium-99. Using this technique, Papagiannopoulou et al. [58] found significant accumulation of the toxin in the lumbosacral DRG after bladder injection in rats.

Other important evidence of axonal transport of peripherally administered BoNTs originate from a number of behavioral and immunochemistry studies focused on sensory system, mainly on nociception and pain. Marinelli et al. [59] analyzed the expression of cl-SNAP25, from the hind paw to the spinal cord, together with the behavioral effects of BoNT/A in a neuropathic pain model. Chronic constriction injury (CCI) of the sciatic nerve in mice was used as an animal model of neuropathic pain, and the effect of an intraplantar (i.pl.) injection of BoNT/A (15 pg/paw from toxin prepared in laboratory) on the neuropathy-induced mechanical allodynia and functional recovery was investigated. It was found that a single i.pl. injection of BoNT/A in neuropathic animals induced long-lasting antiallodynic effects and sped up the functional recovery of injured hind-limbs. Moreover, these behavioral effects correlated with the expression of cl-SNAP25 in tissues along nociceptive pathway, starting from hind paw to sciatic nerve, dorsal root ganglia (DRG), and spinal cord. By immunostaining and confocal microscopy, the expression of cl-SNAP25 was analyzed alone or in colocalization with GFAP, a protein marker expressed in epidermal and hair follicles keratinocytes, in dermal fibroblasts, in non-myelinating Schwann cell and in spinal cord astrocytes. Colocalization of cl-SNAP25 with CD11b, a protein marker of spinal cord microglia, and with NeuN, a marker of neuronal cell nuclei, were also considered. An extensive staining for cl-SNAP25 was observed in all tissue from CCI-induced neuropathic mice treated with i.pl. injection of BoNT/A. Surprisingly, cl-SNAP25 was also detected in spinal astrocytes, suggesting that BoNT/A may be transcytosed from nociceptive fibers in spinal cord to glial cells, confirming that astrocytes express protein involved in vesicular release [60]. The staining of cl-SNAP25 in sections of hind paw, sciatic nerve, DRG, and spinal cord is a strong indication for an axonal transport of BoNT/A along the peripheral nerve to spinal cord. This accounts for BoNT/A trafficking along axonal processes, away from the peripheral site of injection, and transcytosis between neurons and glial spinal cells in the CNS. Axonal retrograde transport of BoNT/A was also observed by Koizumi et al. [61] who found cl-SNAP25 in ipsilateral and contralateral ventral and dorsal horn (DH) in a rat model in which two different subtypes of BoNT/A, namely A1 and A2, were injected ipsilateral into gastrocnemius muscle (1.7–13.6 U/Kg of Botox^®^). The authors showed that serotype A1 was more effective than serotype A2 in spreading, through a transcytosis mechanism, to contralateral spinal cord.

Notable contributions for axonal transport and spinal cord transcytosis of peripheral BoNT/A originate from a series of experiment from Lackovic’s group [62,63,64,65,66,67,68]. In different animal models, Lackovic and colleagues presented much evidence showing that the antinociceptive effects of BoNT/A cannot be fully explained by its peripheral action, and mechanisms of retrograde transport and central transcytosis of peripheral BoNT/A must be considered. In detail, the antinociceptive effects of BoNT/A involving axonal retrograde transport of the toxin was clearly demonstrated in rats subjected to facial pain induced by formalin [64] and to trigeminal neuropathy induced by infraorbital nerve constriction [65]. After injection of BoNT/A (3.5 U/Kg of Botox^®^) into the whisker pad, Matak et al. [64] detected cl-SNAP25 in medullar DH of trigeminal nucleus caudalis. The same authors [66] observed cl-SNAP25 in spinal cord of naive rats after i.pl. or i.m. injection of BoNT/A (5–30 U/Kg of Botox^®^). Colchicine, the microtubule depolymerizing agent blocking the axonal transport, prevents the effects induced by BoNT/A excluding a passive systemic spread of the toxin. Probably, one of the most convincing pieces of evidence was the finding that extracranial injection of BoNT/A (5 U/Kg of Botox^®^ in trigeminal regions) prevents neurogenic inflammation in the cranial dura [67]. This effect was associated with the appearance of cl-SNAP25 colocalized with calcitonin gene-related peptide (CGRP) in neurons innervating the dura mater. It is worth mentioning that the initial impetus to study the effect of BoNTs on pain arose from the empirical observation that many women undergoing cosmetic treatment with BoNT/A for forehead wrinkles, also experienced relief of migraine pain. In vitro patch-clamp studies on substantia gelatinosa neurons of the caudal trigeminal subnucleus, a neuronal system that receives orofacial nociceptive information from primary afferents, confirmed these effects of BoNT/A on the CNS [69]. Strong evidence in favor of retrograde transport of BoNT/A was also presented by Ni et al. [70] who observed an improvement of spatial learning in mice after injection of BoNT/A (2, 10, and 50 U/Kg of Botox^®^) into whisker pads. Retrograde transport was also demonstrated for BoNT/B after unilateral intraplantar delivery [71].

Moving to humans’ studies, Marchand-Pauvert et al. [72] reported experimental evidence supporting the direct central effect of muscular injected BoNT/A (doses not specified) for treatment of spasticity in patients affected by spastic leg paresis, developed after ischemia, hemorrhage, or head injury. They reported a reduction in posterior tibial nerve inhibition of vastus H-reflex following BoNT/A injection in triceps surae muscle. It was hypothesized that the reduction in spinal inhibition would be caused by a modification of the recurrent inhibitory pathway. The reduction in recurrent inhibition, induced by peripherally injected BoNT/A, appears to be a consequence of axonal transport and blockage of the cholinergic synapse between motoneuron recurrent collaterals and Renshaw cells. Similar results were observed by Aymard et al. [73] who proved the modification of reciprocal inhibition of the tibialis anterior muscle mediated by the posterior tibialis nerve after peripheral BoNT injection (doses not specified) in the ankle plantar flexors.

In spite of much evidence presented, the molecular mechanism controlling the axonal retrograde transport of BoNTs, its modulator, and additional cargoes is still far from being understood. One of the major hypotheses is that axonal trafficking of BoNTs could essentially occur through the same mechanism as for axonal transport of tetanus toxin [74,75], with BoNTs sharing the same transport organelles. According to this point of view, axonal retrograde transport of BoNTs occurs in various steps, first involving motoneuron axon. Myelinated motoneurons innervate peripheral muscles via NMJ, while their soma forms contacts with adjacent interneurons and upper neurons located in the spinal cord. When BoNTs are injected into a specific muscle, they are internalized at the NMJ. The majority of BoNTs molecules remain at the NMJ, in which they cleave different SNARE proteins depending from serotype. A fraction of BoNTs may indeed enter organelles targeted to the soma, such as axonal signaling endosomes [55] or by autophagosomes [76], which are transported to the soma in the same way as neurotrophins and their receptors are transported. This long-range axonal transport is performed by cytoplasmic dynein, the microtubule-based motor. Once in the soma of motoneuron, BoNTs could be released into the extracellular medium of spinal cord and internalized by transcytosis into spinal neuron, where again they can target the corresponding SNARE proteins. Although the hypothesized mechanism can explain the observed results, the exact molecular mechanism and cellular components remain to be defined.

## 4. Central Effects of BoNTs after Direct Injection on CNS

Direct injection of BoNTs in the CNS allows us to specifically investigate the role of synaptic activity in different physiological and pathological processes of CNS. The use of BoNTs as tools to block synaptic function in specific regions of spinal cord, brainstem, or brain, can be exploited for therapeutic purposes to counteract pathological hyperactivity diseases in CNS, but also for the basic understanding of CNS functions and of activity-dependent pathways. As BoNTs are toxic substances, for obvious reasons of safety, results presented in this section came almost exclusively from animal models.

Although the peripheral activity of BoNTs were extensively documented, their effects at the level of CNS was never directly investigated in vivo until the 2000s. A few old experiments, performed on cultured cells or animal brain tissue, provided evidence that BoNTs inhibit the release of neurotransmitters in cortical slices and cerebral cortex synaptosomes [77,78,79,80,81,82,83,84]. To our knowledge, we characterized for the first time the in vivo central toxicity and the recovery of health status after intracerebroventricular (i.c.v.) injection of BoNT/A or/B (7.5 and 75 pg/mouse of toxin prepared in laboratory for both serotypes) in mice [85]. This study was propaedeutic to the findings of which sub-lethal doses of the two BoNTs serotype would be useful as experimental tool for in vivo study of cognitive functions, by means of possible functional alteration of neural network induced by BoNTs directly administered into CNS or in specific brain regions. In fact, in a successive study in mice [86] we demonstrated the potential contribution of BoNTs to understand mechanism and/or pathways involved in neuronal processes related to cognitive function. In detail, after i.c.v. injection of sub-lethal doses of BoNT/A or/B (3.75 pg/mouse for both serotypes) in mice, the behavioral responses in conditioning of active avoidance, object recognition test, and pharmacological induced locomotor activity were tested. Compared to control mice, BoNT-treated mice showed a reduced memory in object recognition test, an enhanced stimulant effect of scopolamine, and a depressant effect of oxotremorine, on locomotor activity. In contrast, central injection of the two BoNTs serotypes did not alter active avoidance acquisition. Later, another study [87] showed that a single i.c.v injection of BoNT/A (2 U/Kg of Botox^®^) in rats significantly impaired the water maze performance. Central injections of BoNTs directly in the brain were also used as tool in studies aimed at the comprehension of the mechanisms of pain. In this regard, we performed a comparison between peripheral i.pl. and central i.c.v. injections of BoNT/A and B in the formalin induced inflammatory pain in mice [88]. The main result was that, depending on route of administration and serotype considered, BoNT/A and B (BoNT/A: 0.937–15 pgtox/mouse; BoNT/B: 1.875–7.5 pgtox/mouse) exerted different effects on the behavioral responses induced by the long-lasting nociceptive stimulation of formalin. In detail, BoNT/A inhibited the second inflammatory phase of formalin test, while BoNT/B affected only the interphase between the first acute and the second inflammatory phase of formalin test. This difference was explained considering the different SNARE protein target of the two BoNTs serotypes, SNAP25 for BoNT/A, and VAMP/synaptobrevin for BoNT/B.

Altogether, these results suggested that application of BoNTs into specific brain regions might represent an innovative animal model for in vivo studying the functional alteration of cognitive pathways in several neurological diseases. This gave a strong impetus to studies in which, instead of being injected into the brain’s ventricles, BoNTs were injected intracerebrally into specific brain regions directly involved in cognitive deficits [89]. For example, as it is well established that a loss of cholinergic neurons in the entorhinal cortex is a primary event in Alzheimer’s disease, in vivo injection of BoNT/B (see [90] for doses) into rat entorhinal cortexes have been used to generate a model of dementia with cognitive deficits of learning and memory in maze tests [90]. In another series of studies, Caleo and coworkers used intrahippocampal injections of BoNT/E as tool to evaluate the involvement of hippocampus in spatial learning in the Morris water maze in normal rats and, in the same way, to demonstrate the anticonvulsant and antiepileptogenic properties of BoNT/E (1.5 μL of 50 nM solution of Wako toxin) in a kainic acid induced model of acute seizures and epileptogenesis in rats [91,92,93,94]. Moreover, they demonstrated that intrahippocampal infusion of BoNT/E (0.2 μL of 10 nM solution of Wako toxin) blocked the spike activity of pyramidal neurons by blocking the glutamate release [95]. A reduction in the spontaneous recurrent seizures in a mouse model of temporal lobe epilepsy was also demonstrated [96]. Neuroprotective effects of BoNT/E were also evidenced in a model of focal ischemia induced by infusing the potent vasoconstriction peptide endothelin-1 into the CA1 area of the hippocampus in adult rats [97]. In this model, the injection of endothelin-1 produced a transient and massive increase in glutamate release that was potently antagonized by BoNT/E (1 μL of 25 nM solution of Wako toxin), with a corresponding increase of cell survival in the hippocampus. Similar to BoNT/E, reduced incidence of seizures was also observed after intrahippocampal injection of BoNT/A (subtype A2) in mouse model of temporal lobe epilepsy [98]. Block of seizures in a hippocampal neuronal injury, after induction of an epilepticus status with pilocarpine in rats, was also demonstrated by Huang et al. [99] who administered BoNT/A (150 U/rat of Lanzhou toxin) via intranasal route. These results demonstrated that, under specific conditions of administration, BoNT/A may bypass the blood–brain barrier, suggesting the intranasal administration of BoNT/A as less invasive strategy for neuroprotection in epileptogenesis compared with intracranial injection. In another experiment, Gasior et al. [100] injected BoNT/A or/B (1–10 ng/rat from laboratory prepared toxin) into the rat amygdala to attenuate seizures provoked by electric stimulation of the amygdala. They showed that both BoNTs prevented or attenuated seizures in rats, demonstrating that locally delivered BoNTs can produce prolonged inhibition of brain excitability. Altogether these results suggest a possible use of BoNTs for therapy of neurological disorders that would benefit from suppression of neurotransmission in well circumscribed brain regions.

BoNT/A was also considered as a tool to investigate the involvements of striatum in cognitive and neural determinants of response strategy in a dual-solution plus-maze task in mice [101]. The idea of injecting BoNTs into the striatum paved the way for their possible, still hypothetical, use in the therapy of Parkinson disease (PD), one of the most diffused brain neurodegeneration. It is well known that PD is characterized by loss of dopaminergic neurons in the substantia nigra pars compacta. Disinhibition of tonically active cholinergic interneurons is one of the deleterious consequences of the lack of striatal dopaminergic input. Increased release of ACh by disinhibited cholinergic interneurons results in striatal hyperactivity causing major motor symptoms. Due to pre-synaptic inhibition of ACh release produced by BoNT/A, intrastriatal injections have been considered to improve motor deficits in PD rodent models. Wree et al. [102] observed that intrastriatal injection of BoNT/A (see [102] for doses) abolished pathologic rotational behavior and induces axonal varicosities in the 6-hydroxydopamine (6-OHDA) rat model of PD. In similar experiment, Itakura et al. [103] found that injection of subtype A2 of BoNT/A (0.1, 0.5, or 1 ng/rat of laboratory prepared toxin) into striatum was more efficient in reducing pathogenic behavior compared with subtype A1. Following this line, the research group from the Rostock University performed a long series of investigations on the experimental treatment of striatal cholinergic hyperactivity by injection of BoNT/A into the striatum of rats and mice, focusing on hemi Parkinsonian (hemi-PD) animal models (reviewed in [104]; see also subsequent research in [105,106,107]). They found that, in hemi-PD animals, intrastriatally applied BoNT/A had positive effect on motor dysfunction without impairing cognitive and peripheral cholinergic functions. In similar experiments, amelioration of rotational asymmetry and gait abnormalities was also observed after injection of BoNT/A (0.5 ng of laboratory prepared toxin) in subthalamic nucleus, precisely into the rat entopeduncular nucleus, in 6-OHDA rat model of PD [108,109]. These changes were associated to BoNT/A ability to selectively target glutamatergic terminals.

Recently, in addition to the known improvement of motor performance, an antidepressant-like effect has been demonstrated following intrastriatal injection of BoNT/A (1 ng; two injections of 1 μL solution) in a hemi-PD rat model [110]. Regarding the effects of BoNTs in depression, Ibragic et al. [111] quantified the concentrations of dopamine (DA), noradrenaline (NA), serotonin (5-HT), and their metabolites in brain regions, ipsilateral, and contralateral, from the site of unilateral BoNT/A administration (5 U/Kg of Botox^®^) into the rat whisker pad. From this analysis, authors found a significant increase of NA in striatum and 5-HT in hypothalamus demonstrating an efficacy role for BoNT/A in the treatment of depression. Mann et al. [112] analyzed the densities of dopaminergic (D_1_ and D_2_/D_3_), noradrenergic (α_1_ and α_2_), and serotonergic (5-HT_2A_) receptors in the caudate putamen of the hemi-PD rat model induced by unilateral 6-OHDA injection. In control rats, moderate increase of D_1_ and D_2_/D_3_ densities, together with reduction in 5-HT_2A_ density, were observed, while α_1_ and α_2_ receptor density remained almost unaltered. In rats injected with BoNT/A (1 ng; 2 × 1 μL) a reduction in D_2_/D_3_ receptor density was observed, whereas the densities of the other receptors remained unaltered. Authors concluded that therapeutic effect of BoNT/A on the impaired motor behavior of hemi-PD rats was due to reduction in D_2_/D_3_ receptor density. In another study, Li et al. [113] demonstrated an antidepressant-like effect of single facial injection of BoNT/A (0.06 U and 0.18 U of Lanzhou toxin) in space restriction stressed mice. The effect of BoNT/A was associated with an enhancement of 5-HT level and the expression of brain derived neurotrophic factor in hippocampus, hypothalamus, prefrontal cortex, and amygdala, together with the transiently increased levels of p-ERK and CREB. These preclinical studies, together with some indirect clinical evidence [114,115], suggest BoNT/A as alternative treatment for depression. However, the direct injection of BoNTs to the brain is a procedure that poses ethical and practical difficulties to translate therapeutical benefit from animal model to human. A recent work from Kandasamy’s group [116] found that a mild i.m. injection of BoNT/A (1 U/Kg of Botox^®^) in the thigh of aging mice reduced the level of innate anxiety-related symptoms, measured by the open field, elevated plus maze, and light–dark box tests. Behavioral effects of BoNT/A were paralleled by an increased activities of hippocampal antioxidant enzymes, such as superoxide dismutase and catalase, and reduced glutathione and glutathione peroxidase, compared to the control group. The mechanism of action of BoNT/A in producing such behavioral and enzymatic effects remains unclear.

Regarding the spinal cord, although BoNTs have been considered as an off-label adjuvant therapy in treatment of both spasticity and bladder compliance in spinal cord injured patients [117,118], only a few studies have analyzed the effect of BoNTs directly injected in the spinal cord [24]. Marinelli et al. [119] performed i.th. injection of BoNT/A (15 pg; 5 μL solution of laboratory prepared toxin) in spinal cord of rats subjected to chronic constriction injury (CCI) of sciatic nerve as model of neuropathic pain. It was observed that i.th. injection of BoNT/A counteracts neuropathic pain symptoms induced by CCI, decreasing both mechanical allodynia and thermal hyperalgesia measured on CCI rats’ hind paws. Moreover, intrathecally injected BoNT/A (5 U/Kg of Botox^®^) reduced hyperalgesia in a model of diabetic neuropathic pain induced by streptozotocin peritoneal injection in rats [62], and significantly decreased the nociceptive responses in the formalin test in mice [120]. Interestingly, i.th. BoNT/A (0.01 U/mouse) attenuated the expression level of CGRP, p-ERK, and p-CaMK-II in the lumbar spinal DH compared with control mice [120]. Intrathecal injection of BoNT/A (5 U/rat of Botox^®^) reduced pain symptoms, bladder hyperactivity, expression of neuronal activation markers, c-Fos, p-ERK and GAP43, and CGRP in a rat model of bladder pain and hyperactivity induced by intraperitoneal injection of cyclophosphamide [121]. Moreover, intrathecal application of BoNT/A also significantly reduced the number of abdominal writhes in two other rat models of visceral pain, namely peritonitis and colitis, obtained by intraperitoneal injection of acetic acid or by intracolonic instillation of capsaicin [121], respectively. In the experimental colitis model, BoNT/A reduced both referred mechanical allodynia and c-Fos expression in the DH of the spinal cord. In a model of mirror pain, induced by carrageenan i.m. injection in rats, Drinovac et al. [122] examined the bilateral antinociceptive action after either i.pl. peripheral, both ipsilateral, and contralateral to injury, or i.th. spinal, or intracisternal (i.c.) injection of BoNT/A. They found that i.th. BoNT/A (1 U/Kg of Botox^®^) reduced the bilateral mechanical sensitivity while contralateral i.pl. or i.c. treatments had no effect on both tested sides. Antinociceptive effect of ipsilateral i.pl. BoNT/A (5 U/Kg of Botox^®^) was prevented by μ-opioid antagonist naloxonazine and GABAA antagonist bicuculline only if applied at the i.th. spinal level, in contrast to i.c. supraspinal application. In another study, Coehlo et al. [123] analyzed the effect of i.th. injection of BoNT/A (5 U/rat of Botox^®^) in contrasting the high frequency of voiding contractions and increased intravesical pressure, leading to urinary incontinence, in a rat model of chronic spinal cord injury (SCI). They found that i.th. injection of BoNT/A led to a significant reduction in the frequency of expulsive contractions and a normalization of bladder basal pressure while maintaining voiding contractions of normal amplitude. Cleaved SNAP-25 protein was detected at the DH regions, where most of the bladder afferents end, but not in motor or preganglionic parasympathetic neurons. A significant decrease in CGRP expression occurred both at spinal cord and dorsal root ganglia. Finally, in a model of postoperative pain induced by plantar incision in rats, Li et al. [124] found that i.th. pretreatment with BoNT/A (0.5 U/rat of Botox^®^), induced a prolonged decrease in pain scores and mechanical hypersensitivity. Behavioral effects correlated with reduced expression of SNAP-25 in the ipsilateral lumbar DRG and spinal cord DH, and attenuated increase in NK1 receptor internalization in DH neurons. Antinociceptive effects of BoNT/A were synergically enhanced by i.th. pretreatment with gabapentin.

Additionally, the effect of i.th. injection of BoNT/B was investigated in different pain model [125,126]. Huang et al. [124] found that i.th. pretreatment with BoNT/B (0.5 U/mouse of Myobloc^®^) produced a long-lasting reduction in the release of substance P from spinal afferent nociceptors, the spinal c-Fos expression, and the nociceptive behavior in the model of formalin pain in mice. These effects correlated with BoNT/B cleavage of VAMPI/II protein. In the model of spinal nerve ligation in mice, i.th. BoNT/B attenuated tactile allodynia without effects upon motor function. Interestingly these effects of BoNT/B were not observed in rats, both in formalin or spinal nerve ligation, which is consistent with rat resistance to BoNT/B. Park et al. [126] found that i.th. BoNT/B (0.1–0.5 U/mouse of Myobloc^®^) yields a long-lasting attenuation of the allodynia both in mice displaying mononeuropathy, induced by nerve ligation, or polyneuropathy, induced by treatment with cisplatin.

Recently, the effect of BoNT/A (15 pg/mouse, laboratory prepared toxin) on spinal regeneration and functional recovery after spinal traumas has been analyzed in two model of SCI contusion model [127]. A long-lasting paralysis and pain insensitivity was induced in a severe trauma model useful to evaluate the effects of BoNT/A on motor and sensitivity recovery, axonal regeneration, and neuroprotection. Instead, a short-term reversible paralysis was induced in the moderate trauma model, allowing us to evaluate the effect of BoNT/A on neuropathic pain associated to SCI. In both models, a single dose of BoNT/A was i.th. administered within one hour from contusion. The authors found that BoNT/restores thermal sensitivity and improves motor control in both models of SCI. Moreover, in moderate SCI model, control saline injected mice immediately developed allodynia. On the other hand, SCI mice subjected to i.th. BoNT/A treatement did not develop allodynia. These behaviors were accompanied by a series of cellular, tissue, and functional adaptations, which included: (i) motor neurons reconnection and recovery of muscle atrophy; (ii) reduction in glial cell size and modulation of glia scarring; (iii) preservation of normoglycemic profiles; (iv) protection from cell death and remyelination, with preservation of myelin basic protein (MBP); and (v) stimulation of stem cells production. In summary, i.th. treatment with BoNT/A during the acute phase of SCI reduced tissue damage and promoted motoneurons survival and spinal cord regeneration. Although the comprehension of all molecular events responsible for the spinal regeneration induced by BoNT/A needs to be deeply elucidated, study of Vacca et al. [127] opens a new scenario in therapy of spinal lesions and, as pharmacology, safety, and toxicity of BoNT/A are well documented, strongly encourage the clinical translation.

## 5. Concluding Remarks

Many years passed since Alan B. Scott discovered the potential of BoNTs in medicine and became, for a wide range of pathologies difficult to treat with common drugs, such as dystonia, Parkinsonism, chronic pain, hyperhidrosis, urological dysfunctions, etc., a drug of excellence, perhaps the only one with proven efficacy. Despite this, and despite the numerous experimental evidences of their therapeutic potential obtained in animal models, there still remains a great barrier to their clinical use in the treatment of human CNS pathologies due to cerebral neuronal hyperactivity. Indeed, BoNTs are and remain the most potent natural poison, which, for obvious safety reasons, limits their direct use in the human brain. In reality, this problem is only apparent, and can be circumvented by considering the protein structure of the BoNTs, consisting of two protein domains, one binding and translocating, and one containing the protease for cleavage of the target SNARE proteins. This bi-chain structure offers the advantage of being able to create chimeric proteins with binding and translocation domains specifically designed to reach well-defined sites of action in the CNS, without running the risk of undesirable systemic spread of BoNTs. Much work has been undertaken, and many examples of these chimeric proteins have been produced to ensure that BoNTs can be re-targeted to non-muscular sites [128,129,130,131,132,133,134,135,136,137,138]. Interestingly, a new versatile platform has been recently developed for selective reprogramming of BoNTs protease domain to cleave new targets of therapeutic interest [139]. Although more work is needed before BoNT-based therapies become usable in human CNS pathologies, the way is open, and probably one day, hopefully as close as possible, we may have one more weapon against those human neurological pathologies that are currently lacking truly effective treatments, such as those due to brain disorders and neurodegeneration.
